# New avenues for regulation of lipid metabolism by thyroid hormones and analogs

**DOI:** 10.3389/fphys.2014.00475

**Published:** 2014-12-05

**Authors:** Rosalba Senese, Pasquale Lasala, Cristina Leanza, Pieter de Lange

**Affiliations:** Dipartimento di Scienze e Tecnologie Ambientali, Biologiche e Farmaceutiche, Seconda Università degli Studi di NapoliCaserta, Italy

**Keywords:** thyroid hormones, lipid metabolism, energy balance, insulin resistance development, obesity

## Abstract

Weight loss due to negative energy balance is a goal in counteracting obesity and type 2 diabetes mellitus. The thyroid is known to be an important regulator of energy metabolism through the action of thyroid hormones (THs). The classic, active TH, 3,5,3′-triiodo-L-thyronine (T3) acts predominantly by binding to nuclear receptors termed TH receptors (TRs), that recognize TH response elements (TREs) on the DNA, and so regulate transcription. T3 also acts through “non-genomic” pathways that do not necessarily involve TRs. Lipid-lowering therapies have been suggested to have potential benefits, however, the establishment of comprehensive therapeutic strategies is still awaited. One drawback of using T3 in counteracting obesity has been the occurrence of heart rhythm disturbances. These are mediated through one TR, termed TRα. The end of the previous century saw the exploration of TH mimetics that specifically bind to TR beta in order to prevent cardiac disturbances, and TH derivatives such as 3,5-diiodo-L-thyronine (T2), that possess interesting biological activities. Several TH derivatives and functional analogs have low affinity for the TRs, and are suggested to act predominantly through non-genomic pathways. All this has opened new perspectives in thyroid physiology and TH derivative usage as anti-obesity therapies. This review addresses the pros and cons of these compounds, in light of their effects on energy balance regulation and on lipid/cholesterol metabolism.

## Introduction

The thyroid plays a crucial role in the control of energy metabolism through action of thyroid hormones (THs) in metabolically active tissues such as liver, skeletal muscle and brown adipose tissue. Knowledge on TH action has increased significantly after the cloning of the TH receptors (TRs) termed TRα (including splice-variants α1 and α2 of which α2 does not bind to TH) and TRβ (including transcripts β 1 and β 2, with β 1 predominantly involved in metabolic control, see for a more detailed review: Mullur et al., 2014). TRα1 (henceforth: TRα) is expressed in the brain and to a lesser extent in kidney, skeletal muscle, lungs, heart, and liver, whereas TRβ 1 (henceforth: TRβ) is expressed predominantly in the kidneys and liver, and at lower levels in brain, heart, thyroid, skeletal muscle, lungs, and spleen (Williams, 2000). In absence of hormone, transcriptional regulation through the TRs is blocked through association with co-repressors (Astapova and Hollenberg, 2013). An example comes from a recent study reporting expression of an inactive mutant of nuclear receptor co-repressor (NCoR), NCoRΔID, in mouse liver which, by favoring coactivator recruitment and receptor activation, resulted in increased expression of genes encoding enzymes involved in bile acid metabolism that are under transcriptional control of TRβ (Astapova et al., 2014). Interestingly, one coactivator of TRβ has been recently discovered to be a sirtuin, namely SIRT1 (Singh et al., 2013; Suh et al., 2013; Thakran et al., 2013). To exert their action, TRs commonly heterodimerize with the retinoic X receptor (RXR) but may also homodimerize (Forman et al., 1992). In addition, TRs interact with other nuclear receptors including peroxisome proliferator activated receptors (PPARs), (Bogazzi et al., 1994; Buroker et al., 2007; de Lange et al., 2007). TR-PPAR interactions are of particular importance in regulation of lipid metabolism. The *in vivo* association of unliganded TRα with PPARα has been shown to inhibit PPARα signaling in the liver, a process abolished by T3 (Liu et al., 2007). A dominant negative TRα mutant associated with PPARα in a way that could not be abolished by T3, resulting in hepatic steatosis (Liu et al., 2007). Thus, these results imply that T3 “unblocks” PPARα action by relieving TRα association with PPARα. In accordance, TRα knock-out mice were shown to be protected from diet-induced hepatic insulin resistance (Jornayvaz et al., 2012). TRβ isoforms reduce serum lipids *in vivo* (Johansson et al., 2005; Angelin and Rudling, 2010; Pramfalk et al., 2011; Shoemaker et al., 2012). TRβ disruption in mice has been shown to impair fatty acid oxidation (Araki et al., 2009) which persisted with TRα overexpression (Gullberg et al., 2000, 2002). This implies that T3 increases lipid metabolism both by binding to TRα and TRβ and through PPARs, however through different underlying mechanisms. Physical interactions between TRβ and PPARα in mouse heart (Buroker et al., 2007), and PPARδ in rat skeletal muscle (de Lange et al., 2007) have been suggested to increase expression of common target genes involved in lipid metabolism.

## The development of specific TRβ agonists as lipid/cholesterol-lowering agents

Hyperthyroidism can lead to thyrotoxicosis, with increased heart rate, atrial arrhythmias and heart failure, muscle wasting, post- menopausal osteoporosis in women, fatigue, anxiety, and preference for decreased temperatures (Webb, 2004). Basic studies have revealed that TRα is responsible for TH's effects on heart rate: mice lacking TRα showed reduced heart rate and body temperature (Wikström et al., 1998), and echocardiograph studies in mice deficient in TRα or TRβ have revealed that the effects of THs on heart rate are TRα-dependent (Weiss et al., 2002). Cardiac contractile functions and the expression of genes involved therein have been shown to be all TRα-dependent (Gloss et al., 2001).

Initial studies on TH analogs date from before the cloning of the TRs and showed that analogs binding strongly to hepatic TRs but weakly to heart TRs (Ichikawa et al., 2000) were effective in reducing body weight and serum lipid/cholesterol levels but did not cause evident cardiac arrhythmias. L-94901, the first described selective thyromimetic compound with 50% of the binding affinity of T3 to hepatic TRs and only a minimal affinity to cardiac TRs (Underwood et al., 1986), has been reported to lower plasma cholesterol in experimental animals without inducing cardiotoxic side effects. A subsequent compound, CGS 23425, lowered total serum and LDL cholesterol and increased synthesis of apolipoprotein A1 (APOA1). Indeed, after the cloning of the TRs, CGS 23425 was confirmed to be weakly TRβ-selective (Taylor et al., 1997).

Because of TRα-associated unwanted side effects of TH, the nineties saw the development of TRβ-specific ligands [these comprise GC-1 (Chiellini et al., 1998), its derivative GC-24 (Borngraeber et al., 2003), and KB141 (Grover et al., 2005)], or, alternatively, ligands targeted to the liver. The first such cholesterol-reducing compound with high liver specificity was CGH-509A (derived from conjugation of L-T3 and cholic acid). Other drugs developed for targeting to the liver were MB-07811, a prodrug of the active metabolite MB07344 (Erion et al., 2007) and KB-2115 (Berkenstam et al., 2008). Several compounds that were intentionally designed to be liver-specific were subsequently also found to be TRβ-selective, and *vice versa*. For instance, the selective TRβ-agonist GC-1 has been found to be preferentially taken up by the liver (Baxter and Webb, 2009) and, in turn, MB-07811 resulted to be more than ten-fold TRβ-selective. TRβ affinity varies between compounds: GC-1 (Chiellini et al., 1998) has equal TRβ affinity with respect to T3, GC-24 (Borngraeber et al., 2003) and MB-07344 (Erion et al., 2007; Fujitaki et al., 2008) have a relative twofold-lower affinity, and KB-141 has a sixfold lower relative affinity (Erion et al., 2007). In addition, the synthetic agonists have different pharmacokinetic and pharmacodynamics properties with respect to T3 (Erion et al., 2007). Finally, effective tissue uptake differs between the compounds, which could be related to their relative affinities for the classic TH transporters such as monocarboxylate transporter-8 (MCT-8) (Erion et al., 2007).

## TH analogs and cholesterol metabolism

A large number of TH analogs ameliorate lipid and lipoprotein metabolism through the lowering of plasma total and LDL cholesterol levels and the stimulation of reverse cholesterol transport (RCT) in different animal models (Baxter and Webb, 2009; Tancevski et al., 2009; Pramfalk et al., 2011). The LDL cholesterol-lowering effect exerted by TRβ1- and liver-selective thyromimetics such as KB-141, GC-1, KB-2115, MB-07811, T-0681, CGS-23425, and DITPA (Underwood et al., 1986; Taylor et al., 1997; Grover et al., 2003, 2004; Erion et al., 2007; Tancevski et al., 2009) is ascribed to increased hepatic LDL clearance via stimulation of LDL receptor (Erion et al., 2007; Tancevski et al., 2009, 2010) and the stimulation of cholesterol 7 alpha-hydroxylase (CYP7A1) expression and activity (Gullberg et al., 2000; Johansson et al., 2005; Erion et al., 2007; Tancevski et al., 2010) enhancing hepatic cholesterol uptake and its conversion into bile acids to be excreted by feces. Moreover, T0681 induces the expression of ABCG5 and ABCG8, a tandem pump promoting the biliary excretion of free cholesterol (Tancevski et al., 2010). Recent mechanistic studies in mice showed the LDL receptor (LDLr) expression to be crucial for the hypocholesterolemic effects of MB-07811 and for T-0681 (Erion et al., 2007; Tancevski et al., 2010) which was not the case for that of T3, GC-1 and KB-2115 (Goldberg et al., 2012; Lin et al., 2012). These data suggest that patients with familial hypercholesterolemia may benefit from a treatment with specific TH analogs.

## TRβ agonists and prevention of hepatic steatosis: side effects including insulin resistance

At first the use of TRβ agonists to ameliorate lipid profiles was considered unfavorable due to their potential causing, in analogy with T3, of adipose lipolysis, and induction of hepatic lipogenesis and thus steatosis. In fact, T3 poorly reduces hepatic steatosis in rodent obesity models (Cable et al., 2009). Genetically obese/diabetic rodent models or models of rodents placed on high-fat diets have increasingly been used to investigate the efficiency of these compounds to lower lipid profiles and to counteract non-alcoholic fatty liver disease (NAFLD) (Cable et al., 2009; Vatner et al., 2012) and hepatic insulin resistance (Vatner et al., 2012), with varying outcomes.

MB-07811 efficiently reduced hepatic steatosis as well as plasma FFA and triglycerides in various rodent models including male ob/ob mice, male Zucker diabetic fatty (ZDF) rats, and male C57Bl/6 mice placed on a high-fat rodent diet (60% fat by kcal) for 3 months. This compound, in contrast to T3, did not cause adipose lipolysis, and efficiently reduced hepatic steatosis by inducing hepatic fatty acid oxidation and mitochondrial respiration rates, phenomena known to be related to hepatic TR activation. Unlike T3, MB-07811 did not increase heart weight and neither did it decrease pituitary thyroid-stimulating hormone beta (TSHβ) expression (Cable et al., 2009). Additional TRβ agonists, namely GC-1 and KB-2115, tested in similar systems have proven to be effective in depleting liver lipids, (Cable et al., 2009; Vatner et al., 2012), but recent evidence has shed doubt on the potential use of these compounds to ameliorate insulin sensitivity (Vatner et al., 2012). Male Sprague-Dawley rats treated daily with GC-1 while being placed on a commercial high-fat diet showed a 75% reduction of hepatic triglyceride content, but developed fasting hyperglycemia and hyperinsulinemia due to increased glucose production and diminished hepatic insulin sensitivity. In addition, white adipose lipolysis was increased, which the authors suggest to contribute to endogenous glucose production due to the increased glycerol flux (Vatner et al., 2012). Rats being fed the same diet period but treated daily with KB-2115 displayed a reduction of hepatic steatosis without evident fasting hyperglycemia, increased glucose production or diminished hepatic insulin sensitivity. Instead, insulin-stimulated muscle glucose uptake was diminished with concomitant reductions in glucose transporter 4 (GLUT4) protein content (Vatner et al., 2012). The induction of insulin resistance at various levels by several TRβ agonists may put their therapeutic potential into question. Nevertheless, KB-2115 recently entered phase III clinical trials which were discontinued when cartilage damage was observed in a long-term study on dogs (Sjouke et al., 2014).

Despite these drawbacks, the need to develop agents that counteract dyslipidemia persists, thus the search for effective TRβ agonists remains justified. An overview of the metabolic effects of the described compounds is given in Table 1.

**Table 1:** **From thyromimetics to TRβ ligands and TH metabolites: reported TRβ affinities and metabolic effects**.

**Ligand**	**Displays TRβ binding affinity (with respect to T3)**	**Reduces Plasma LDL cholesterol**	**Acts LDL receptor- dependent**	**Prevents hepatic steatosis**	**Causes insulin resistance**	**Increases heart weight/rate**
**THYROMIMETIC**						
L-94901	Weak	Yes				No
CGS-23425	Weak	Yes				No
**TRβ AGONIST**						
GC-1	Equal	Yes	No	Yes	Yes	No
GC-24	2-fold lower	Yes	Yes	Yes		No
KB-141	6-fold lower	Yes	Yes			No
T-0681		Yes	Yes			No
DITPA		Yes	Yes			No
**LIVER-TARGETED**						
CGH-509A		Yes				No
MB-07811		Yes		Yes		No
MB-07344	2-fold lower	Yes		Yes		No
KB-2115		Yes	No		Yes	No
**TH METABOLITE**						
TRIAC	3-fold higher however no (or weak) TRβ specificity					No
T1AM	None	No	No		Yes	No
T2	60-fold lower (human TRβ) with no TRβ specificity	Yes	No	Yes	No (rats)	No/No (rats)
					ND (mice)	Yes/ND (mice)^*^

Abbreviations: ND, not determined;

* *dose of T2 used in mice is 10-fold higher*.

## Action of T3 and other TH metabolites by not directly interacting with nuclear TR-TREs

In recent years, it has become ever more clear that T3 exerts its effects not only through nuclear TR-TRE interactions, but also through cytosolic TRs or even independent of TRs. Ten percent of the TR pool is localized within the cystosol (Baumann et al., 2001) and T3 has been shown to interact with these cytosolic TRs to influence phosphatidyl inositol 3- kinase (PI3K)-Akt signaling, with phosphorylated Akt subsequently influencing transcription of genes involved in glucose metabolism via mammalian target of rapamycin (mTOR) (Moeller et al., 2006). T3-induced phosphorylation of Akt in rat skeletal muscle causes glucose transporter 4 (GLUT4) translocation to the sarcolemma (de Lange et al., 2008). Truncated forms of TRs are also present in mitochondria. A variant of TRα in mitochondria (Wrutniak-Cabello et al., 2001) has been suggested to directly stimulate oxidative phosphorylation upon interaction with T3 (Oetting and Yen, 2007).

Other receptors have been shown to interact with T3: (Davis et al., 2005). TH interacts with integrin alpha V beta 3 in the cell membrane. This event triggers the MAPK/ERK pathway with phosphorylated MAPK translocating into the nucleus and associating with TRβ (Plow et al., 2000). This causes phosphorylation of the TRβ receptor, enhancing its action on transcription rate (Davis et al., 2000). Thus, TR activation can be enhanced through pathways other than solely TH binding.

**Figure 1:**
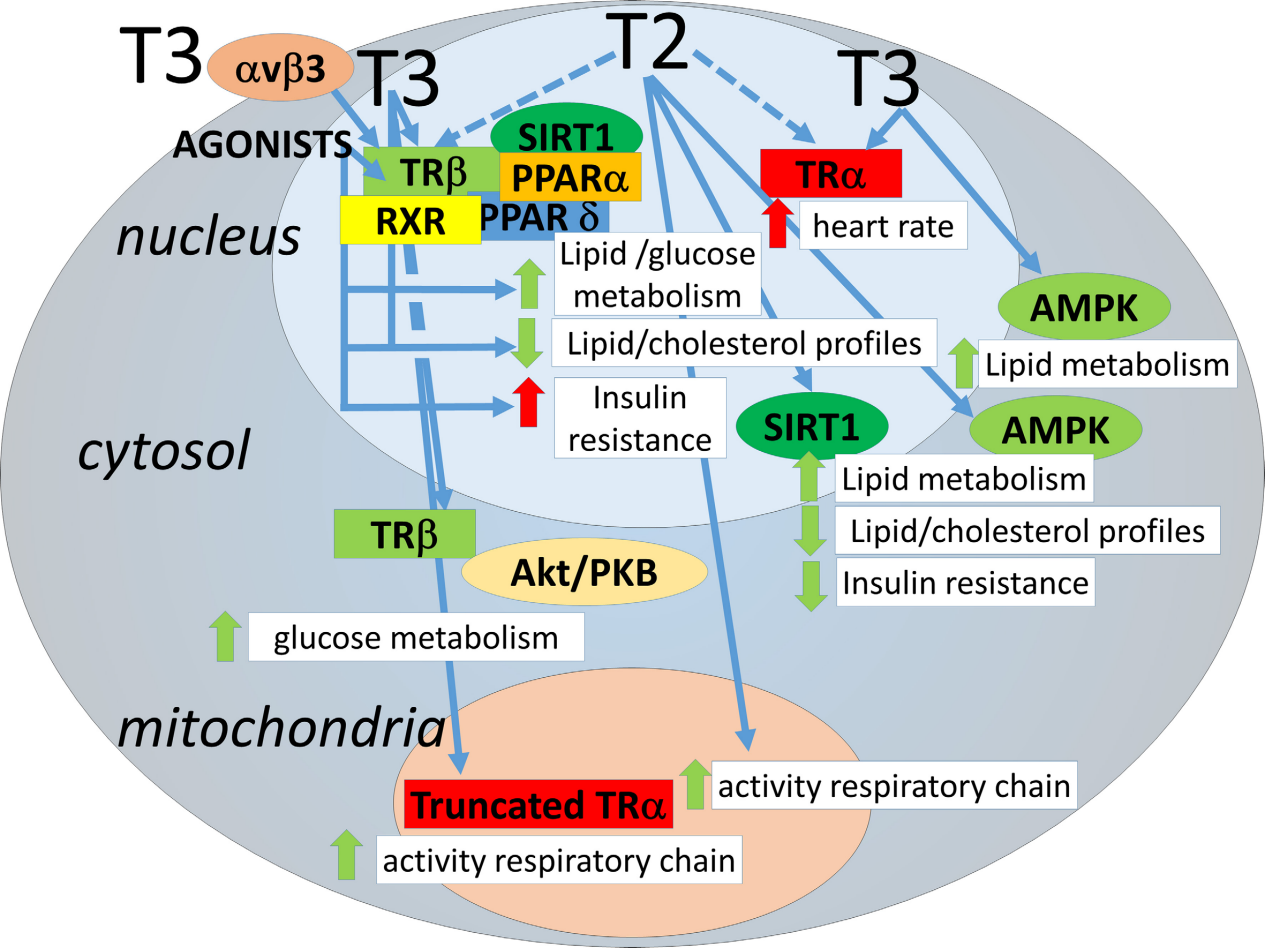
**Avenues for regulation of lipid metabolism by thyroid hormones and analogs**. THs may act through transcriptional regulation by nuclear TR binding but also through interactions involving cytosolic/mitochondrial TRs or other proteins, each of which influencing lipid metabolism. For clarity, the TH analogs have not been depicted separately, negative effects on insulin sensitivity have only been demonstrated for a subset (see text). Of the thyroid hormone metabolites only T2 is depicted, since T2 is the only TH metabolite thus far with low affinity for TRα and positive effects on metabolism. Avenues are depicted as blue arrows. Dotted arrows: yet to be determined/confirmed. Effects are depicted as red arrows (negative) or green arrows (positive).

Furthermore, T3 causes activation of non-receptor proteins. AMP-activated kinase (AMPK), which plays a central role in lipid and glucose metabolism homeostasis (Hardie et al., 2012), has been shown to be a target of transient and rapid activation (within hours) by T3 in skeletal muscle (de Lange et al., 2008; Irrcher et al., 2008). Sirtuins are NAD+ activated deacetylases that have been shown to play a role in metabolic homeostasis (Chang and Guarente, 2014). T3 has been shown to activate SIRT1 by various groups, and this depends on interaction with TRβ (Singh et al., 2013; Suh et al., 2013; Thakran et al., 2013). Some effects of T3 through SIRT1 require binding of TRβ to TREs (Suh et al., 2013; Thakran et al., 2013). However, one target of T3-TRβ-SIRT1 action is FOXO1, which upon deacetylation triggers the expression of genes involved in gluconeogenesis in mice that do not necessarily contain a TRE in their promoters (Singh et al., 2013). Some of the above mentioned mechanisms of action exerted by T3 are shared by other TH metabolites. AMPK activation in skeletal muscle has been found also to occur by 3,5-diiodo-L-thyronine (T2) (Lombardi et al., 2009), as well as SIRT1 activation in liver (de Lange et al., 2011) and in kidney (Shang et al., 2013). These results have added to the realization that some of the actions of T3 (including those on energy metabolism) can overlap with those of the TH metabolites with low affinity for TRs. The mechanistic aspects of the metabolic effects of the thyroid hormone analogs and metabolites are highlighted in Figure 1.

## Natural TH metabolites, promising lipid-reducing agents?

Besides T3, the thyroid is a source of other iodothyronines, which are either produced within the thyroid itself or are deiodinated periferically, the so-called “non-classical” THs (see for an more extensive overview Senese et al., 2014). One non-classical TH with high TR affinity, Triac (3,5,3′-triiodothyroacetic acid), has been shown to be weakly TRβ-selective and to lower cholesterol without reduced effects on heart rate (Moreno et al., 2008). Other TH metabolites, due to their weak binding to TRs, do not predominantly act through binding to nuclear TRs and by modulating TRE-mediated gene transcription. An example is T1AM, an amine present in serum of rodents and humans (Saba et al., 2010; Hoefig et al., 2011), and in rat liver and brain (Saba et al., 2010). T1AM has no affinity for TRβ and TRα (Chiellini et al., 1998). T1AM, however, is a potent agonist of trace amine-associated receptor 1 (TAAR1), an orphan G protein-coupled receptor (GPCR) (Zucchi et al., 2006). Contrary to T3, T1AM does not ameliorate lipid profiles and may cause insulin resistance depending on the dose: intracerebroventricular (icv) injection of T1AM into short-term fasted male mice in a dose of 130 ng/100 gBW (Manni et al., 2012) causes hypophagia as well as peripheral effects namely raised plasma glucose levels and reduced peripheral insulin sensitivity despite increased pancreatic insulin production. This would classify this compound as unsuitable for improving metabolic profiles. Another TH metabolite which has been under study in recent years is T2. This compound has a 60-fold reduced binding capacity to human TRβ (Mendoza et al., 2013). In line with this, it has been shown that T2 has a weak transactivating capacity of TRβ target genes in different systems, *in vitro* and *in vivo* (Ball et al., 1997; Cioffi et al., 2010; de Lange et al., 2011; Mendoza et al., 2013). Interestingly, T2 has an affinity for TRβ in fish that is similar to that of T3 (Mendoza et al., 2013), indicating an evolutionary role for this iodothyronine as a genuine classic TH. In Wistar rats housed at thermoneutrality and placed on a high-fat diet (50% fat), T2 (25 μg/100 g BW) induces a protein profile favoring a shift toward fast-twitch, type II skeletal muscle fibers, and a preference for glucose as fuel (Moreno et al., 2011). Under the same conditions, T2 has strong lipid lowering effects and can effectively prevent hepatic steatosis, ameliorating tissue and systemic insulin sensitivity (de Lange et al., 2011). T2, apart from binding directly to subunit Va of mitochondrial cytochrome-c oxidase, enhancing its activity (Arnold et al., 1998), acts by activating hepatic nuclear SIRT1, which deacetylates PGC-1α and SREBP-1c, thus inducing the expression of genes involved in mitochondrial fatty acid oxidation, and repressing genes involved in lipogenesis, respectively, confirmed by proteomic profiling (de Lange et al., 2011). Thyroid hormones, ameliorating metabolic parameters, may improve healthy aging (see for review de Lange et al., 2013). Indeed, very recently, Padron et al. (2014) reported that T2 administration (25, 50, and 75 μg/100 g BW) to aging Wistar rats reduced body- and retroperitoneal fat mass gain, increased resting metabolic rate (RMR), ameliorated glucose tolerance, did not alter heart mass and heart rate, and only lowered serum T4 and T3 levels at the two higher doses (Padron et al., 2014). A similar effect was recently observed in HFD-fed mice which showed increased heart weights only when treated with unusual very high doses of T2 (250 μg/100 g BW) (Jonas et al., 2014). Reaching a “safe” dose of T2 may thus be feasible in humans as well, which warrants further investigation.

## Conclusions and perspectives

There has been much progress in identifying “novel” mechanisms of action of T3 and T2 (Figure 1) and TH metabolites and in developing “safe” (that is: non-thyrotoxic) TH-related compounds to reduce lipid profiles. The discovery of thyroid-related compounds which could be implied in the regulation of energy balance may thus pave the way to strategies for their use in the clinic.

### Conflict of interest statement

The authors declare that the research was conducted in the absence of any commercial or financial relationships that could be construed as a potential conflict of interest.
